# A Technique for Removing Implant-Retained Denture: Direct Relining Complication

**DOI:** 10.1155/2015/126257

**Published:** 2015-06-23

**Authors:** İbrahim Duran, Betül Yilmaz, Çağrı Ural

**Affiliations:** Department of Prosthodontics, Faculty of Dentistry, Ondokuz Mayıs University, 55139 Samsun, Turkey

## Abstract

The aim of this case report is to present a technique for removing the denture which locked to ball-attachment because of excessive hard relining resin material flows around the ball-attachment. An alternative method was used in the present case. A cylindrical resin was removed with a diamond bur at the level of matrix and by this way the matrix was removed safely. The advantage of the presented method is that it may be extended to other clinical situations when facing a similar complication for implant supported dentures and also that the technique is simple and does not require special equipment.

## 1. Introduction

An implant-retained complete dental prosthesis can be a viable and effective treatment option for patients who are dissatisfied with conventional dentures. This type of prosthesis comes with necessary maintenance requirements [1]. Continuing bone resorption leads to a poor fit and a lack of support of the denture base. Therefore these kinds of prosthesis may require periodic relining to reestablish tissue support for the denture base. There are 2 main methods of relining dentures: the direct (chairside) method and the indirect or processed method. Some autopolymerizing acrylic resins have been developed specifically as hard chairside reline resins for relining dentures directly in the mouth [2, 3] and these materials have been found to be very successful in improving the retention and stability of removable prostheses [4].

In direct hard relining concept the denture was relined chairside with cold-curing acrylic resin material. Relining undercuts in the sulcular areas should be blocked out using rubber dam and plastic rings placed around the abutments [5].

Nevertheless there is a risk that the relined denture cannot be removed from the mouth, if the excess reline resin flows and polymerizes into undercuts of abutment teeth, soft tissue, or implant components. For this or similar reasons the implant-retained dentures must be removed from the mouth with minimal trauma to denture, implant components, and gingival tissue. This case report presents a technique for removing the denture which locked to ball-attachment because of excessive resin polymerized.

## 2. Case Report

**Figure 1:**
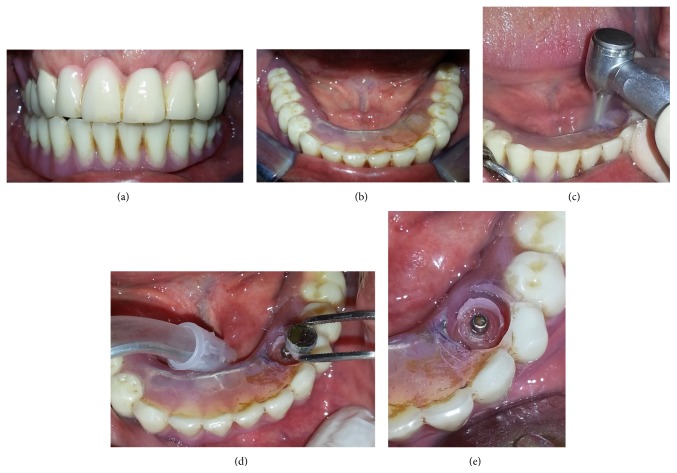
(a) Intraoral view of the prosthesis, (b) View of the mandibular implant supported denture, (c) marking the relevant portion of the denture, (d) removing matrix from the mouth, (e) implant supported denture with removed matrix.

**Figure 2:**
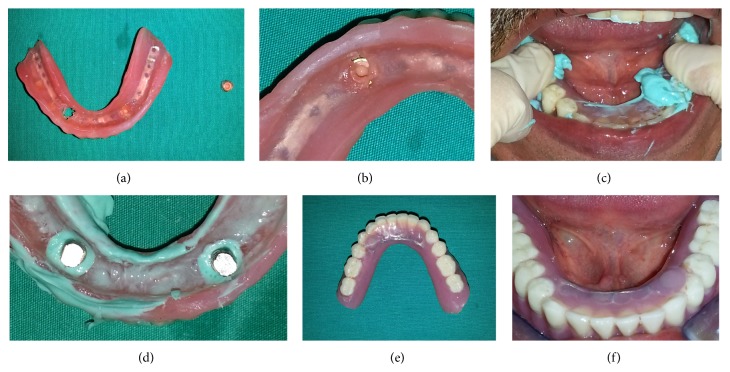
(a) View of the removed matrix, (b) view of the excessive resin material, (c) making a new impression with light impression material, (d) placing the implant analogs, (e) view of the repaired denture, (f) placing the repaired denture to the mouth.

48-year-old male patient was referred to our clinic (Department of Prosthodontics) regarding a complication in his lower two implant supported dentures (Figures 1(a) and 1(b)). The relining procedure had been done directly in the patient's mouth using hard relining material by a dentist in private office. The denture could not be removed from the mouth and the reason was probably the excessive resin between the matrix and ball-attachments. Specifically, there was no movement on the left of the denture and thus we started the process from left matrix. Removing the matrix safely is the primary purpose of this technique. To this and the relevant portion of the denture was marked with indelible pencil (Figure 1(c)). A cylindrical resin was removed with a diamond bur at the level of matrix and by this way the matrix was removed safely (Figures 1(c) and 1(d)). And then the excessive resin around the ball-attachment was removed with a conical diamond bur. After the process the denture was removed by pulling out gently (Figure 1(e)). The denture and left matrix are seen in Figures 2(a) and 2(b). Previously made hard relining material was removed from the denture's internal and external surfaces. To perform the indirect relining technique and repairing the denture, light silicon impression material was applied to the inner surface of the denture and when placed in mouth patient was advised to close the mouth without pressure to avoid displacement of the soft tissues (Figure 2(c)). After laboratory procedures performed, the denture, occlusion, borders, and soft tissue adaptation were evaluated. The final image of the repaired denture was seen in Figures 2(e) and 2(f).

The aim of this case report was to present a technique for removing the denture which locked to ball-attachment because of excessive hard relining resin material flows around the ball-attachment. The advantage of the presented method is that it may be extended to other clinical situations when facing a similar complication for implant supported dentures and also that the technique is simple and does not require special equipment.

## 3. Discussion

Garrett et al. [6] found that almost all patients perceived improvement in chewing comfort, chewing ability, less difficulty eating hard foods, and eating enjoyment. Most patients also reported improvements in speech and security. These results support the beliefs of clinicians and observations of some researchers that patients benefit from properly fitting dentures so the reline procedure is most often used when factors other than loss of bone or soft-tissue support have changed for the patient (i.e., the vertical dimension, occlusion, phonetics, and functionality of the dentures are acceptable). For the implant-retained dentures direct relining, acrylic resin barriers must be used to protect the undercuts before prosthesis relining [5]; otherwise such complications can occur because of excessive hard relining resin material flows around the ball-attachment. The present technique showed a conservative solution to clinicians when they are faced with this kind of problem.

Although this approach could be of low cost and beneficial treatment alternative for both upper and lower implant supported dentures, further cases with different kinds of implant components and more representative cases should be conducted to validate the performance of this alternative method.

## References

[1] Davis D. M., Packer M. E., Watson R. M. (2003). Maintenance requirements of implant-supported fixed prostheses opposed by implant-supported fixed prostheses, natural teeth, or complete dentures: a 5-year retrospective study. *International Journal of Prosthodontics*.

[2] Arima T., Murata H., Hamada T. (1995). Properties of highly cross-linked autopolymerizing reline acrylic resins. *The Journal of Prosthetic Dentistry*.

[3] Urban V. M., Machado A. L., Vergani C. E. (2009). Effect of water-bath post-polymerization on the mechanical properties, degree of conversion, and leaching of residual compounds of hard chairside reline resins. *Dental Materials*.

[4] Haywood J., Basker R. M., Watson C. J., Wood D. J. (2003). A comparison of three hard chairside denture reline materials. Part I. Clinical evaluation. *The European Journal of Prosthodontics and Restorative Dentistry*.

[5] Romanos G. E., May S., May D. (2011). Treatment concept of the edentulous mandible with prefabricated telescopic abutments and immediate functional loading. *The International Journal of Oral & Maxillofacial Implants*.

[6] Garrett N. R., Kapur K. K., Perez P. (1996). Effects of improvements of poorly fitting dentures and new dentures on patient satisfaction. *Journal of Prosthetic Dentistry*.

